# Histologic tissue response to furcation perforation repair using mineral trioxide aggregate or dental pulp stem cells loaded onto treated dentin matrix or tricalcium phosphate

**DOI:** 10.1007/s00784-016-1967-0

**Published:** 2016-10-20

**Authors:** H Bakhtiar, H Mirzaei, M R Bagheri, N Fani, F Mashhadiabbas, M Baghaban Eslaminejad, D Sharifi, M H Nekoofar, PMH Dummer

**Affiliations:** 1 Dental Material Research Center, Tehran Dental Branch, Islamic Azad University, Tehran, Iran; 2 Department of Stem Cells and Developmental Biology, Cell Sciences Research Center, Royan Institute for Stem Cell Biology and Technology, ACECR, Tehran, Iran; 3 Department of Oral and Maxillofacial Pathology, School of Dentistry, Shahid Beheshti Medical Science University, Tehran, Iran; 4 Department of Surgery and Radiology, Faculty of Veterinary Medicine, Tehran University, Tehran, Iran; 5 Department of Endodontics, School of Dentistry, Tehran University of Medical Sciences, Tehran, Iran; 6 Endodontology Research Group, School of Dentistry, College of Biomedical and Life Sciences, Cardiff University, Cardiff, UK

**Keywords:** Bioactive scaffold materials, Regeneration, Stem cells, Treated Dentin matrix (TDM), Tricalcium Phosphate (TCP)

## Abstract

**Objectives:**

The aim of this study is to compare the effect of treated dentine matrix (TDM) and tricalcium phosphate (TCP) scaffolds on odontogenic differentiation and mineralization of dental pulp stem cells (DPSCs) in furcation perforations created in the pulp chamber floor of premolar teeth in dogs.

**Material and methods:**

DPSCs were isolated and cultured from the dental pulp of the maxillary left second and third premolars of dogs. The DPSCs were loaded on TCP (SC+TCP) and TDM (SC+TDM) scaffolds and inserted into intentionally perforated pulp chamber floors of premolars in dogs; six teeth were used for each group. Three more groups of six specimens were created, and mineral trioxide aggregate (MTA), TDM, and TCP were inserted into the perforations to act as controls. An intact premolar and no treatment in the perforation site were used as positive and negative controls respectively. After 3 months, the animals were sacrificed and the type of inflammation, presence of dentine, continuation and type of cementum, type of connective tissue, and presence of foreign body reaction were evaluated, and significant differences were between groups determined using the Fisher’s exact test. The evaluation of the amount of inflammation and the percentage of new bone formation was evaluated using the Mann-Whitney *U* test.

**Results:**

The negative control group was associated with severe inflammation and granulation tissue formation. In the positive control group, intact periodontal tissues and no inflammation were observed. Dentine bridge formation was not seen in specimens of any group. The specimens in the SC+TDM group were associated with significantly more bone formation than other groups (*P* < 0.001). The amount of inflammation was less than 10 % in specimens of all groups with the exception of three specimens in the TCP group that were categorized as 10–30 %. Chronic inflammation without foreign body reactions was the major pattern of inflammation in groups. Formation of cementum with a cellular and continuous appearance was seen in all specimens.

**Conclusions:**

SC+TDM was associated with significantly more bone formation when used to repair uninfected furcation perforations in the premolar teeth of dogs.

**Clinical relevance:**

Application of TDM as a biological scaffold in combination with DPSCs may offer an advantage during the repair of root perforation defects.

## Introduction

Furcal perforations are the consequence of procedural errors or pathologic processes, such as caries or root resorption, and can affect the outcome of root canal treatment. The outcome of teeth with perforations depends on the size, location of the defect, the time before repair, and the degree of periodontal irritation [1]. A wide range of materials including mineral trioxide aggregate (MTA) can repair perforation sites [2]. Indeed, new cementum has been reported to form adjacent to MTA without inflammation [3, 4]. However, MTA is nondegradable [5] and unable to be replaced by natural tissue [6]. Moreover, in a large furcal perforation, it is difficult to control the material and can extrude into the surrounding periodontal ligament space, triggering tissue inflammation and a foreign body reaction [7]. Thus, repair of root perforations remains a clinical challenge.

Regenerative medicine is based on stem cells, growth factors, and scaffolds [8]. Mesenchymal stem cells (MSCs) are used for cell-based treatments for a variety of tissue defects due to their capacity for replication and multilineage differentiation. These cells were first isolated and described from bone marrow [9] but are also present in dental pulp tissue [10].

Dental pulp stem cells (DPSCs) are classified as postnatal or multipotent stem cells [6]. It has been demonstrated that DPSCs can differentiate into odontoblast-like cells and have the capacity to secrete dental matrix at the site of injury. Among the three main components of regeneration medicine (stem cells, growth factors, and scaffolds), stem cells are believed to be the most important contributing factor. In addition, scaffold is required to organize the cells and create a tissue construct [11].

Tricalcium phosphate (TCP) is a degradable bioceramic material suggested for use as a scaffold for human dental pulp cells because it delivers statin, which is able to aid their differentiation into odontoblasts [11]. Moreover, treated dentine matrix (TDM), that is, dentine components treated with ethylenediaminetetraacetic acid (EDTA), has been shown to stimulate reactionary dentinogenesis in nonexposed cavity preparations and tooth defects [12–14]. EDTA treatment provides a reliable source of transforming growth factor-β1 (TGF-β1) on dentine surfaces [15]. Therefore, TDM could create a suitable scaffold and an inductive microenvironment for dentine regeneration [8].

MTA is currently the gold standard material for repairing perforations and is able to stimulate the proliferation of human DPSCs [16] and the odontogenic differentiation of DPSCs [17].

The aim of this study was to investigate whether scaffolds consisting of TCP and TDM had the capacity to induce differentiation of DPSCs in furcation perforation defects of dogs in order to produce osteodentine-like tissue. The null hypothesis was that there is no difference among various scaffolds in odontogenic differentiation of DPSCs.

## Materials and methods

### Animal preparation and tooth extraction

The study was undertaken according to the principles of the laboratory animal care of the faculty of Veterinary Medicine, University of Tehran. Power analysis was calculated to support the number of samples per group; therefore, the study was committed to the principles of Replacement, Reduction and Refinement (3Rs). Five 2-year-old mixed-breed dogs with an average weight of 20–25 kg were used. The animals were housed for 1 week to become acclimatized. Under general anesthesia with a combination of xylazine (20 mg/10 kg) (LIDXY, Alfasan, Woerden, Netherlands) and ketamine (30 mg/10 kg) (Quality Pharma Pvt. Ltd., Tamil Nadu, India). Second and third maxillary premolars in each dog (two teeth total per dog) were extracted, and stem cell isolation was performed.

### Isolation and culture of DPSCs

Each tooth was sectioned at the cementoenamel junction (CEJ), and the pulp tissue was retrieved. The tissue was dissected into small pieces and predigested in collagenase (3 mg/mL)/dispaze (4 mg/mL) enzyme (Sigma-Aldrich, Munich, Germany) for 30 min at 37 °C and transferred to a culture plate with 3-mL Dulbecco’s modified Eagle’s medium (DMEM) (Gibco, Carlsbad, CA, USA) supplemented with 15 % bovine fetal serum and 1 % antibiotic mean penicillin (100 U/mL) and streptomycin (100 mg/mL), and centrifuged at 400*g* for 5 min. The pellet was then suspended in fresh medium, plated in a six-well culture plate, and incubated in an atmosphere of 5 % carbon dioxide at 37 °C. The culture medium was changed twice a week, and cells from the third passages were used.

### Cell characterization

#### Multilineage differentiation

For chondrogenic differentiation 2.5 × 10^4^ cells from the third passage of DPSCs were pelleted at 400*g* for 5 min. DMEM supplemented with 10 ng/mL transforming growth factor-β3 (Sigma), 10 ng/mL BMP-6 (Sigma), 50 mg/mL insulin transferrin selenium premix (Sigma), 1.25 mg bovine serum albumin (Sigma), and 1 % FBS were added to the pellets. The cultures were maintained for 3 weeks, during which the medium was changed twice a week. A number of differentiated pellets were prepared histologically, cut into 5-mm-thick sections and stained with toluidine blue for metachromatic matrix detection.

For osteogenic differentiation, 1 × 10^5^ DPSCs were seeded into a six-well plate. At 80 % confluence, the cells were cultured in osteogenic medium containing DMEM supplemented with 50 mg/mL ascorbic 2-phosphate (Sigma, St Louis, MO, USA), 10 nM dexamethasone (Sigma), and 10 mM glycerol phosphate (Sigma) for 3 weeks. During this period, the culture medium was exchanged twice a week. The cultures were then stained with Alizarin Red for mineralized matrix.

For adipogenic differentiation, the confluent cultures were treated with differentiation-inducing medium that consisted of DMEM supplemented with 50 mg/mL ascorbic acid 3-phosphate, 100 nM dexamethasone, and 50 mg/mL indomethacin. After 3 weeks, the cultures were examined by Oil Red O staining for lipid droplets. During the differentiation period, the culture medium was exchanged twice a week.

#### Flow cytometry analysis

Flow cytometry analysis was performed to characterize cells in terms of their surface epitopes. The third passage of stem cells were treated with trypsin and used for flow cytometry analysis. Further, 250,000 cells (counted) were incubated 4 °C and in the dark, with specific antibodies CD90 (BIO Science BD) (Becton, Dickinson and Company, 1 Becton Drive, Franklin Lakes, NJ), CD45 (BIO Science BD), CD44 (BIO Science BD), and CD145 (BIO Science BD) in distinct pipes for 30 min. They were then washed with 1 mL phosphate-buffered saline (PBS) supplemented with 1 % fetal bovine serum (FBS) and centrifuged at 400*g* for 5 min. The cell pellet was then suspended in 300–500 μL of the same solution and analyzed by flow cytometry (FACSCalibur cytometer equipped with 488-nm argon lasers; Becton Dickinson, Franklin Lakes, NJ, USA). Data analysis was undertaken with WinMDI 2.9 software (en.Bio-soft.net/WinMDI.html, miscellaneous free software).

### Scaffold preparation

The premolar teeth were instrumented using a curette to remove the periodontal ligament along with the outer cementum and part of the dentine. Pulp tissue and the predentine layer were also mechanically removed using K-files (Mani, Utsunomiya, Tochigi, Japan). The resulting dentine specimens were divided in two segments. For the fabrication of the TDM, the samples were cleaned mechanically using an ultrasonic cleaner (Blue Wave Ultrasonic, Davenport, IA, USA) and then treated with 17 % EDTA (Sigma, Gaithersburg, Germany) for 5 min, 10 % EDTA for 5 min, and 5 % EDTA for 10 min. This process was repeated three times. TDM were maintained in sterile PBS with 100 UI/mL penicillin (Hyclone, Logan, UT, USA) and 100 mg/mL streptomycin (Hyclone, Logan, UT, USA) for 72 h, then washed in sterile deionized water for 10 min in an ultrasonic cleaner, and then finally stored in DMEM at 4 °C. Morphological observations of TDMs were performed using a scanning electron microscope (SEM) (Zeiss, Munich, Germany) to survey if the size and appearance of porosities that were created on the surface of the TDM were suitable for cell adhesion.

### Cell seeding

Prior to cell seeding the TCP and TDM scaffolds were soaked in DMEM medium in a 24-well plates. A total of 2.5 × 10^5^ cells (third passage) were suspended in 0.2 mL DMEM and placed on the top surface of 2-mm blocks of TDM and TCP. Cells were allowed to attach to the biomaterials for 2 h at 37 °C before adding DMEM. All 3D cultures were maintained in a humid atmosphere at 37 °C and 5 % CO_2_ for 48 h before implantation. Further, the cells penetrated the porous TDM scaffolds and became attached to the TCP ceramic scaffolds. To calculate the number of cells successfully loaded into the scaffolds, all cells that appeared within the wells, either floating or adhered, were collected and counted. To ensure cell attachment, the cell-loaded scaffolds were prepared for observation with a SEM. The combination of cells and scaffolds were then kept in DMEM in a portable incubator until implantation.

### Scanning electron microscopy

MSC-loaded scaffolds (day 2) were fixed in 2.5 % glutaraldehyde at 4 °C for 24 h and then washed with PBS. The samples were dehydrated sequentially with increasing concentrations of ethanol (30, 50, 80, and 100 %), coated with gold, and visualized at an accelerating voltage using a scanning electron microscope (Zeiss, Munich, Germany).

### Creation of perforations in pulp chamber floors

Mandibular second, third, and fourth premolars in each of two quadrants and the maxillary right second and third premolars were used. After general anesthesia, rubber dam application and disinfection of the crown with chlorhexidine 0.12 % (Kin Gingival, Alpantha®, Barcelona, Spain), standard coronal access cavities were prepared using a straight carbide fissure bur (Tizcavan, Tehran, Iran).

The root canals were shaped with Reciproc® size 25, 0.08 taper instruments (VDW, Munich, Germany), and filled with laterally compacted gutta-percha (Dentsply Maillefer, Ballaigues, Switzerland) and AH26 sealer (Dentsply DeTrey, Konstanz, Germany). Then, a 2-mm-diameter perforation was created in the floor of each pulp chamber using a no. 2 round long-shank carbide bur (Jota, Ruthi, Switzerland) in a high-speed handpiece with water coolant (Contra-Angel, BienAir, Bienne, Switzerland) until hemorrhage was noted. The width and depth of all perforations were standardized to the diameter of the size two round burs. The depth of the perforation varied with the dentine-cementum thickness in the furcation area. The area was then dried with compressed air and sterile cotton pellets.

### Perforation repair

Thirty-two premolars in five dogs were randomly divided into five experimental and two control groups:

Group A: Mineral trioxide aggregation (ProRoot tooth colored MTA, Dentsply Maillefer) (*n* = 6)
Group B: Treated dentine matrix (TDM) (*n* = 6)
Group C: TCP (*n* = 6)
Group D: TDM scaffold impregnated with DPSCs (stem cell + TDM) (*n* = 6)
Group E: TCP scaffold impregnated with DPSCs (stem cell + TCP) (*n* = 6)

#### Group A

MTA powder was mixed on a glass slab with distilled water in a 3:1 ratio (powder/water). When the mixture lost its shiny appearance, approximately 30 s after mixing, it was immediately placed into the perforation with an MTA gun (Densply Tulsa Dental, Tulsa, OK, USA). A wet cotton pellet was used to condense the MTA gently into the perforation site. A thin layer of degradable collagen barrier (Collacet, Tokyo, Japan) was inserted above the MTA and the access cavities were then filled with amalgam (SDI, Bayswater, Victoria, Australia).

#### Group B and group C

TDM and TCP were used to repair the perforations, respectively. The size of the TDM and TCP blocks were approximately similar to the perforation sites in order to fill the defects with the exception of a small space for insertion of the collagen barriers on top of the TDM and TCP. The TDM and TCP blocks were inserted gently in the defects using pliers, followed by the collagen and the amalgam (SDI, Bayswater, Victoria, Australia).

#### Group D and group E

DPSCs seeded on TDM scaffolds and TCP scaffolds were placed in the perforation sites. They were picked up gently from the storage media using pliers and inserted into the perforation sites. The perforation repairs were subsequently covered with a barrier of collagen and then amalgam (SDI, Bayswater, Victoria, Australia).

The positive control group was an intact tooth and the negative control group was a tooth without any materials placed in the perforation site and left open.

### Postoperative care

The dogs were kept inside the Faculty of Veterinary Medicine, University of Tehran, under supervision and with continuous monitoring and food intake. After 3 months, vital perfusion was performed with 10 % buffered formalin (Merck Millipore, Billerica, MA, USA).

### Histological preparation and examination

Bone blocks containing the relevant teeth were obtained and placed in 10 % formalin for 2 weeks. Then, the specimens were rinsed for 10 min and placed into 10 % formic acid at ambient temperature for decalcification. The bone blocks were then dehydrated in ascending concentrations of alcohol (from 70 to 100 %) and embedded in paraffin. Finally, five 5-μm-thick sections were prepared with a focus on similar perpendicular cutting angle and evaluated. The sections containing the middle part of the furcation were subjected to hematoxylin and eosin (H&E) staining. The specimens were investigated by an oral pathologist in a blinded manner under an optical microscope (Nikon E400, Japan).

The following parameters were examined:

- The amount of inflammation by counting the ratio of inflammatory cells in 100 cells under the high-power magnification (×400):
  - Score 0: <10 %
  - Score 1: 10–30 %
  - Score 2; 30–50 %
  - Score 3: >50 %
- Type of inflammatory cells: chronic inflammatory cells were the expected cells. The presence of acute cells revealed procedural errors and the specimens were excluded.
- Dentin, i.e., the presence or absence of dentine bridges and type of dentine (osteodentine or regular dentine).
- The type of cementum (cellular or acellular).
- The continuity of cementum (complete or incomplete).
- Type of connective tissue (fibro vascular or granulation tissue).
- Foreign body reaction with the presence or absence of macrophages or giant cells.
- The percentage of new bone formation detected using Nikon 8400 camera and then counting the percentage of bone formation using Iranian histomorphometric (HMM) version 1 software (Sbmc, Tehran Iran).

### Statistical analysis

The Fisher’s exact test was used to analyze the type of inflammation, presence of dentine, continuation of cementum, type of cementum, character of connective tissue, and presence of foreign body reaction. The evaluation of the amount of inflammation and the percentage of new bone formation was undertaken using the Mann-Whitney *U* test.

## Results

### DPSC culture

**Fig. 1:**
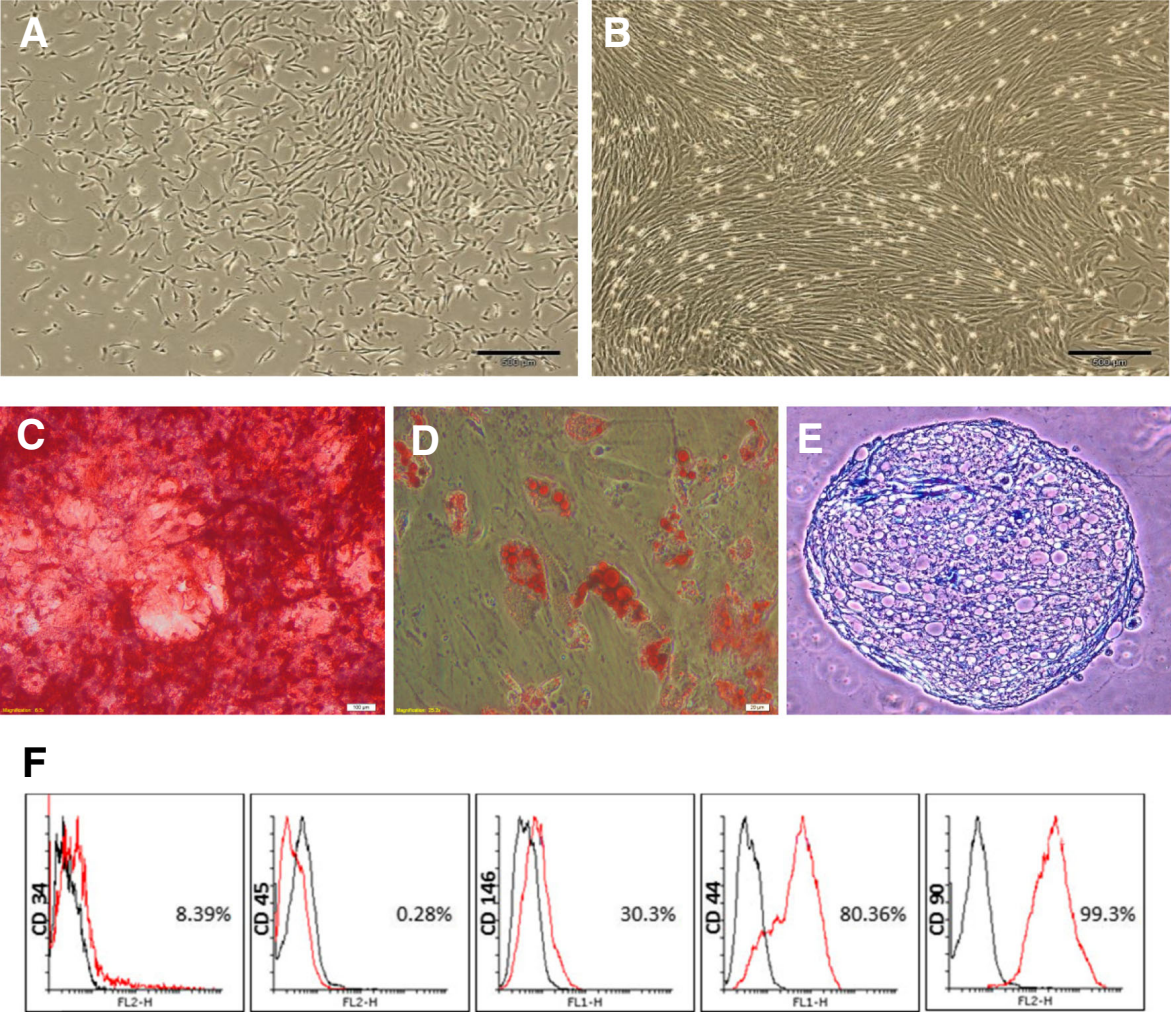
Dental pulp stem cells (DPSCs). **a** In primary cultures, the cells formed large colonies that consisted of fibroblastic cells (*bar* = 200 μm). **b** The colonies increased in size, becoming confluent (*bar* = 200 μm); the *shiny dots* were other cells that are beside dental pulp cells in pulp chamber and cultured with them. **c** Osteogenic differentiation of DPSCs stained by Alizarin Red (*bar* = 100 μm). **d** Adipose differentiation of DPSCs stained by Oil Red O (*bar* = 20 μm). **e** Cartilage differentiation of DPSCs stained by toluidine blue (*bar* = 100 μm). **f** The cells had a surface antigenic profile similar to those of mesenchymal stem cells. While endothelial and hematopoietic markers were present in a very low percentage of the cells, the mesenchymal markers were expressed by the majority of the cell population. *FITC Z* fluorescein isothiocyanate, *PE Z* phycoerythrin

Seven to 10 days after the initiation of the primary culture, several large colonies of fibroblastic cells were detected which increased in size and then became confluent. The cells maintained their spindle-shaped morphology during the passages (Fig. 1a, b).

### Multilineage differentiation

The sections prepared from chondrogenic pellets were metachromatic as pictured by Toluidine Blue staining. Third passage DPSCs succeeded in differentiating to bone cells since the culture treated by osteogenic medium tended to positively stain red with Alizarin Red. In the adipogenic culture, a lipid droplet appeared in the differentiating cell. Positive staining of the globule by Oil Red in the induced cells was the proof of their adipogenic differentiation (Fig. 1c–e).

### Flow cytometric analysis

The majority of the DPSCs tended to express surface markers of MSCs such as CD90 and CD44. Endothelial and hematopoietic cell markers such as CD34 and CD45 were expressed in a low percentage of the isolated cells (Fig. 1f).

### SEM

**Fig. 2:**
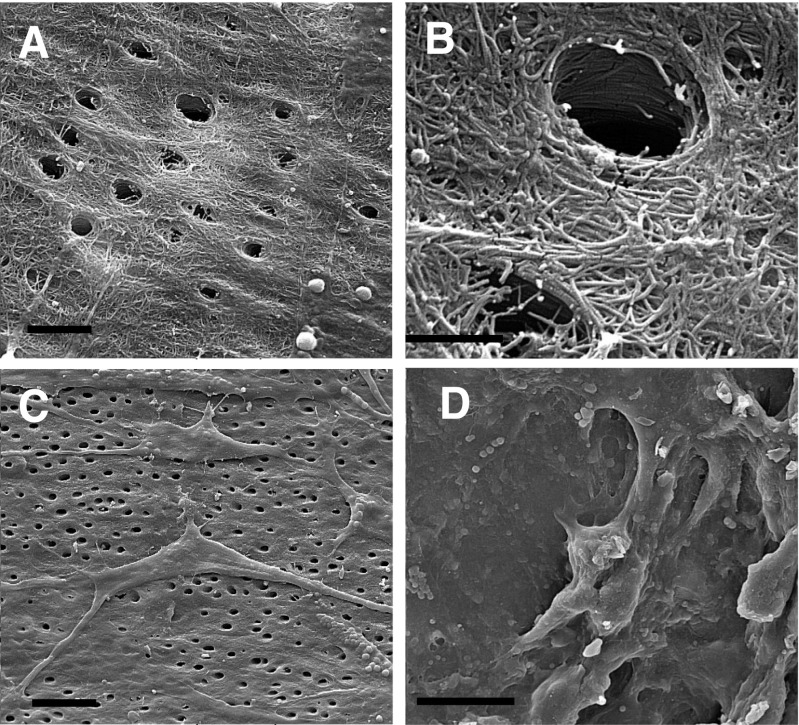
Scanning electron microscopy (SEM) of dental pulp stem cells (DPSCs) loaded onto tricalcium phosphate (TCP) and treated dentine matrix (TDM) scaffolds. TDM scaffold without cells (**a** *bar* = 5 μm, **b** *bar* = 2 μm). **c** TDM with cells (*bar* = 20 μm). **d** TCP with cells (*bar* = 20 μm)

Based on the SEM observations of the scaffold/cell constructions, DPSCs appeared to occupy the scaffold pore spaces in TCP and on the surface of TDM. In the representative images shown in Fig. 2, the cells were seemingly established and attached to the scaffold surfaces (Fig. 2).

### Scaffolds

#### Histologic and histomorphometric results

**Fig. 3:**
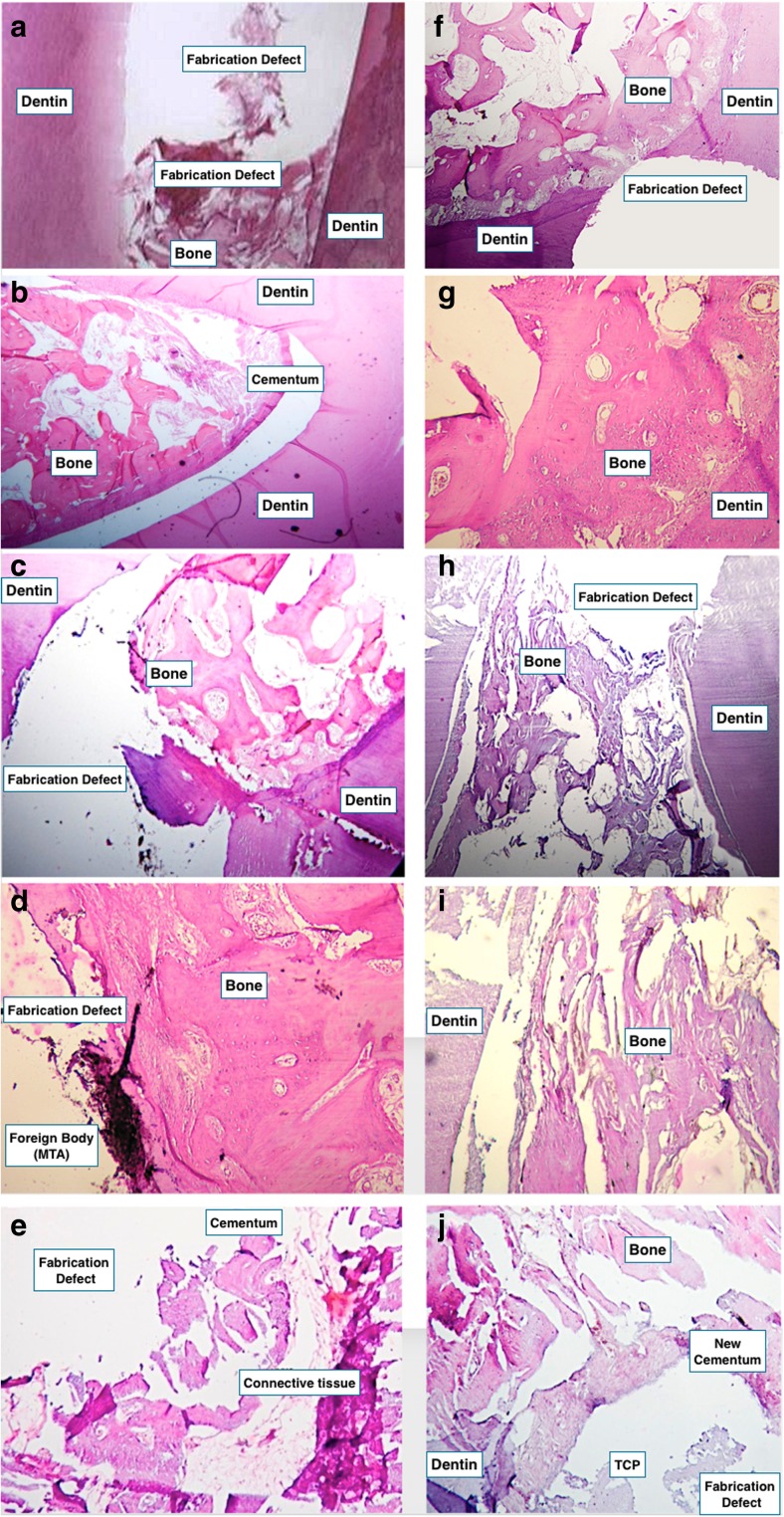
**a** Negative control group; **b** positive control group; **c** MTA group (×40); **d** MTA group (×100); **e** TDM group (×40); **f**, **g** TDM+DPSC group that shows new cementum and compact new bone formation on perforation site (×40, ×100); **h**, **i** TDM+DPSCs group from a different slide (×40, ×100); **j** TCP+DPSCs, H&E stain (×40) (note: the gap between the samples and dentin seen in these images is a processing artifact)

Histopathologic evaluation of the sections in the negative control group revealed severe inflammation (more than 50 %) with granulation tissue formation (Fig. 3a). In the positive group, there was no inflammation and an intact periodontium with fibers was seen (Fig. 3b).

**Fig. 4:**
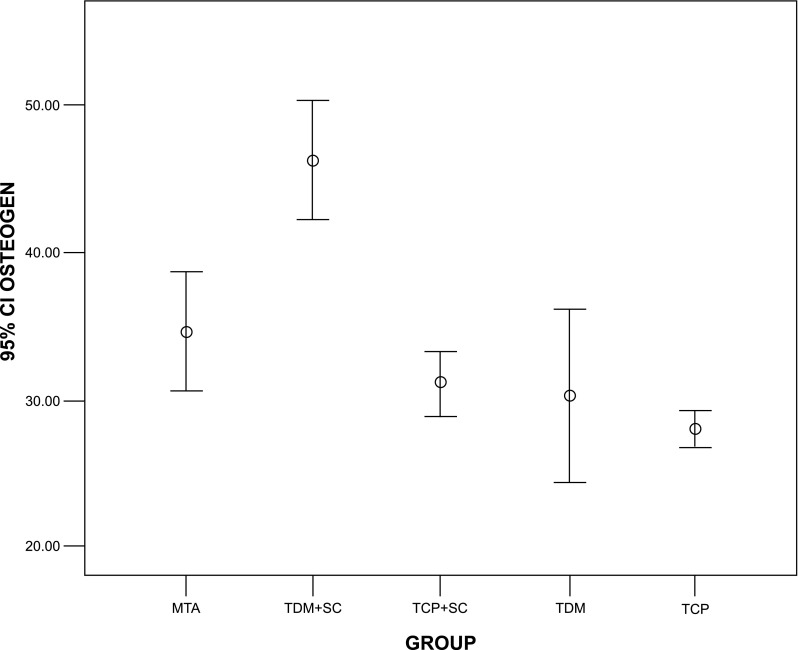
The mean percentage of bone formation at the depth of 50 μm

Evaluation of the mean percentage of new bone formation at a depth of 50 μm under the experimental materials in groups MTA, stem cell + TDM, stem cell + TCP, and TDM and TCP were 34.63 ± 3.18, 46.21 ± 3.26, 31.19 ± 1.72, 30.27 ± 4.79, and 27.99 ± 1.02 %, respectively (Fig. 4). Specimens of the stem cell + TDM group were associated with significantly more bone formation than specimens of the other groups (*P* < 0.001). There was no significant difference between the other experimental groups (Fig. 4).

The amount of inflammation was less than 10 % in all experimental groups with the exception of three specimens of the TCP group, which were in the 10–30 % category. There were significant differences between the inflammation associated with TCP and other groups (*P* < 0.05). Chronic inflammation was the major pattern of inflammation in all groups with no evidence of dentine bridge formation or foreign body reactions. Formation of cementum with a cellular and continuous appearance was seen in all specimens. Vascular connective tissue in PDL was the dominant appearance of connective tissue in all specimens (Fig. 3c–g).

## Discussions

MTA has been suggested as a repair material for perforations located in the floor of pulp chambers [18], even though it is a nondegradable biomaterial and thus unable to induce natural tissues to grow into the perforation site. In the present study, the regenerative potential of DPSCs loaded on TDM and TCP, or pure TDM and TCP, were compared with MTA for the repair of artificially created defects. The results of the present study indicate that a combination of DPSCs and TDM may provide a suitable alternative for regenerative therapy. In addition, the difference between the TDM and DPSC+TDM groups was significant (*P* < 0.001), which indicates that the presence of DPSCs effectively increased the ability of the material to regenerate tissue. This result ruled out our null hypothesis of equal impact of TDM and TCP on differentiation of DPSCs.

The two-rooted dog premolars, which were used in this study, have bifurcations close to the CEJ (1–2 mm). Therefore, this experimental model that produces favorable results in epithelialization and regeneration of furcation perforations is likely to have comparable responses in human [19].

The size of perforations is considered as one of the factors that determine the long-term outcome of furcal perforation repairs. In this study, to keep the size of perforations consistent, a no. 2 round long-shank carbide high-speed bur (Jota, Ruthi, Switzerland) was used to create a 2-mm-diameter perforation in the floor of the pulp chamber. However, due to inconsistencies in the thickness of dentine/cementum at the furcation site, the surrounding tissues including bone may have been damaged.

Stem cells are one of the key factors in regeneration procedure. In the present study, DPSCs were seeded on two different scaffolds, TDM and TCP. Gue et al. [20] suggested autogenous acellular dentinal matrix as a suitable scaffold for dental tissue engineering because of its lack of immunogenicity, ideal mechanical properties, and high concentration of inductive factors for regeneration of dentine. Dentine matrix includes collagen, noncollagenous proteins ,and growth factors, which are essential for remineralization and regeneration of dentine [12]. Furthermore, a combination of EDTA and ultrasonic can effectively remove the smear layer of dentine to produce TDM [21, 22]. It has been demonstrated that 10 % EDTA could influence cellular events involved in dentine repair and regeneration by releasing dentine matrix bioactive components such as isoforms of TGFβ [23, 24]. However, the over demineralization of dentine may result in ineffective scaffolds [12]. Pang et al. [25] showed that when DPSCs were placed in direct contact with EDTA-treated dentine surfaces, it could induce cell attachment and odontoblastic/osteoblastic differentiation. They suggested that EDTA was beneficial for achieving successful outcomes in regenerative endodontics. In the present study, in order to produce treated dentine matrix, a three-step use of various concentration of EDTA combined with ultrasonic treatment was used [25].

DPSCs were isolated from premolar teeth, and their surface antigens were characterized prior to transplantation. Surface markers of MSCs including CD90 and CD44 were used to ensure that the cells belonged to the MSC population [26]. Karamzadeh et al. [27] also used endothelial and hematopoietic cell markers, including CD34 and CD45, to confirm that isolated cells were not contaminated with other cells. The present results show that the majority of the DPSCs tended to express surface markers of MSCs such as CD90 and CD44 (Fig. 1f).

TCP is a degradable bioceramic that has been used as a scaffold for DPSCs [28]. Tada et al. [29] suggested that the chemical composition of TCP with high concentration of calcium and phosphate could increase bone morphogenic protein-2 (BMP-2) expression through cyclic adenosine monophosphate–dependent protein kinase and extracellular signal-regulated kinase 1/2 pathways in human DPSCs.

The histological findings of the present study revealed that the highest and the lowest mean values of new bone formation were associated significantly with specimens of DPSCs seeded on TDM (stem cell + TDM) and pure TCP specimens, respectively (*P* < 0.001). In comparison to conductive scaffolds such as TDM, conventional cell scaffolding materials like TCP provide good structural support for cells yet provide an environment conducive for cell differentiation [20]. In terms of bone formation, there were significant differences between the MTA group and the DPSC+TDM group (*P* < 0.001). Even though MTA is able to assist human mesenchymal stem cell (hMSC) adhesion, growth, and migration [16, 30, 31], a combination of DPSCs and TDM may provide a suitable alternative for regenerative processes in future studies. The difference between the TDM and DPSC+TDM groups was significant (*P* < 0.001), which indicates that the presence of DPSCs effectively increased the ability of the material to regenerate tissue. These results are in agreement with Yang et al. [32] who suggested TDM could be used as a biological scaffold for dental follicle cells (DFCs). DFCSs, under the effect of TDM, highly expressed dentine matrix protein-1 (DMP-1) and bone sialoprotein (BSP), indicating their potential for odontogenesis and osteogenesis [32].

It has been also demonstrated that the microenvironment of the alveolar fossa in combination with TDM could be a very suitable inductive environment for tooth root construction. Gue et al. [33] demonstrated that DFCs are appropriate for seeding stem cells and that TDM can provide both a suitable inductive microenvironment and serve as a good scaffold for dentinogenesis [20, 33]. It has also been documented by previous reports that TDM serves as an odontogenic microenvironment [34].

On the contrary, in the present study, osteogenic regeneration was seen more than dentineogenesis in all specimens. Graziano et al. [35] and Karamzadeh et al. [27] demonstrated that DPSCs have more osteogenic potential than dentinogenic potential to explain why osteogenic regeneration occurred. This finding could also be the result of the site where the cells were embedded as they were surrounded by bone, and it has been shown that autograph tooth treated with EDTA resulted in effective bone formation [36, 37]. For the future studies, application of the scaffolds to various environments is suggested. Since the thickness of the dentine-cementum in the furcation area is inconsistent, in this study, a 2-mm artificial penetration by the bur may have penetrated the periodontal ligament and bone tissue. Indeed, the surrounding bone tissue is more likely to facilitate osteogenesis induction rather than dentineogenesis. According to the histological findings, the continuity of cellular cementum was also superior in the DPSC+TDM group compared with other groups. These findings are aligned with Chen et al. that showed the formation of cementum as part of the periodontium on the exterior of TDM [34]. Considering more than 100 published papers, there is a huge attempt to produce techniques for processing of bone graft material using extracted teeth and it could be anticipated developing scaffolds from homogenous and xenogenous tooth and dental restorative materials using extracted teeth in the near future [38].

## Conclusion

Due to its regenerative potential, TDM could be used as a biological scaffold in combination with DPSCs for repair of endodontic defects. However, a more detailed understanding of mechanisms involved in tooth root regeneration is essential.
